# Supplementary material for: Saccharomyces boulardii (CNCM I-745) improves intestinal damage in sepsis by remodeling intestinal flora structure
Source: Front Cell Infect Microbiol. 2025 Jul 31;15:1602792. doi: 10.3389/fcimb.2025.1602792 (PMC12350329; doi:10.3389/fcimb.2025.1602792)
Supplement: Supplementary file 1 [file DataSheet1.zip › summary/summary/7_Differential_analysis/2_LEfSe_result/Group_AW4_vs_AW0_0.05_3.0/LEfSe_Group_AW4_vs_AW0.abund.pdf]

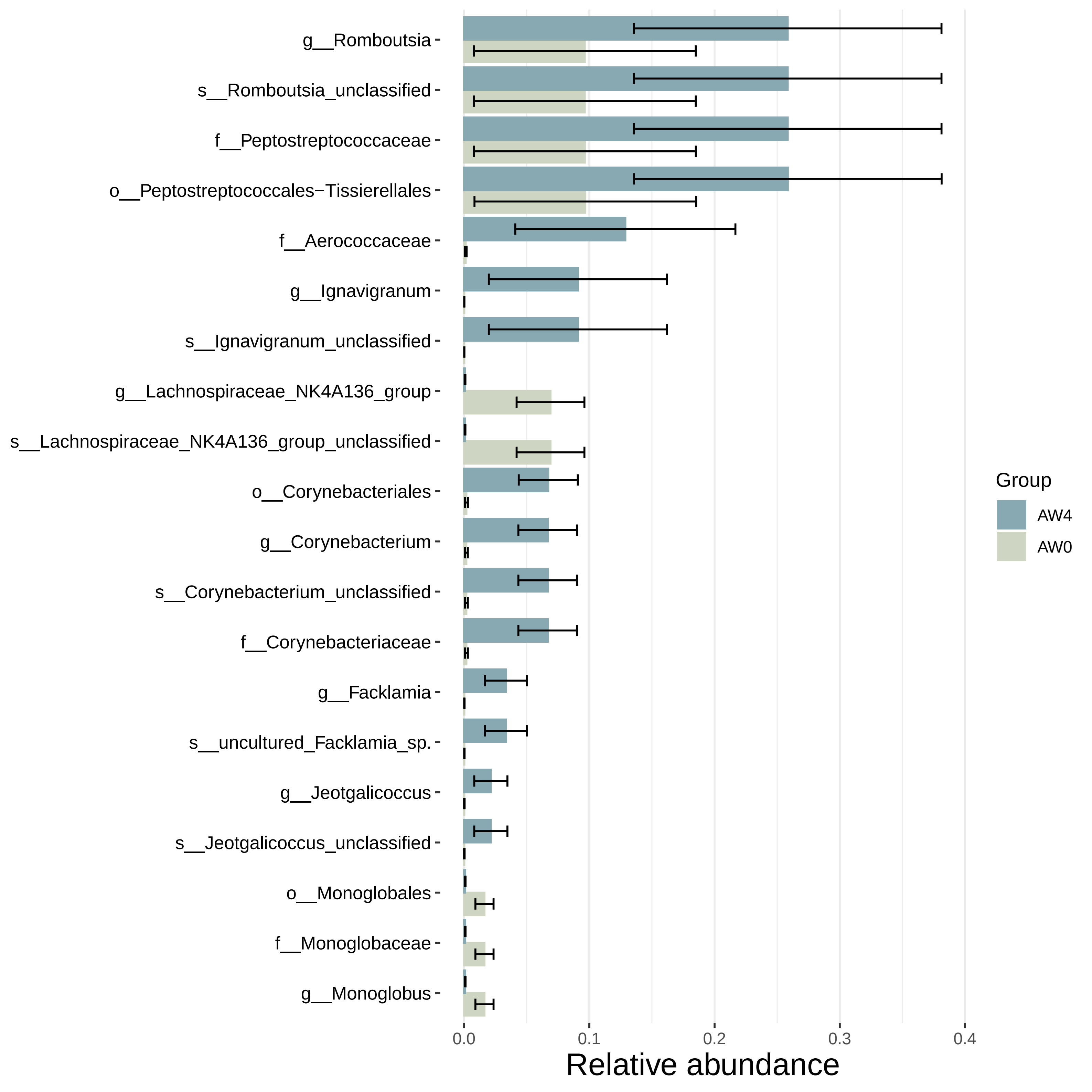

g__Romboutsia
s__Romboutsia_unclassified
f__Peptostreptococcaceae
o__Peptostreptococcales−Tissierellales
f__Aerococcaceae
g__Ignavigranum
s__Ignavigranum_unclassified
g__Lachnospiraceae_NK4A136_group
s__Lachnospiraceae_NK4A136_group_unclassified
o__Corynebacteriales
g__Corynebacterium
s__Corynebacterium_unclassified
f__Corynebacteriaceae
g__Facklamia
s__uncultured_Facklamia_sp.
g__Jeotgalicoccus
s__Jeotgalicoccus_unclassified
o__Monoglobales
f__Monoglobaceae
g__Monoglobus
0.0
0.1
0.2
0.3
0.4
Relative abundance
Group
AW4
AW0
